# Editorial: Exploring Neuroendocrine Mechanisms in Psychiatric and Metabolic Comorbidities

**DOI:** 10.3389/fendo.2026.1801881

**Published:** 2026-02-18

**Authors:** Santosh Chokkakula, Balaji Pathakumari, Ge Hong, Bing Yang

**Affiliations:** 1 Department of Microbiology, Chungbuk National University College of Medicine and Medical Research Institute, Cheongju, Republic of Korea; 2 Department of Medicine, Mayo Clinic, Rochester, MN, United States; 3 State Key Laboratory of Advanced Medical Materials and Devices, Tianjin Key Laboratory of Biomedical Materials, Institute of Biomedical Engineering, Chinese Academy of Medical Sciences and Peking Union Medical College, Tianjin, China; 4 Department of Cell Biology, College of Basic Medical Sciences, Tianjin Medical University, Tianjin, China; 5 Department of Public Health, International School, Krirk University, Bangkok, Thailand

**Keywords:** mental disorders, metabolic comorbidities, metabolic health, neuroendocrine mechanisms, psychiatric

The human body functions as a highly integrated biochemical network governed by intricate regulatory and feedback mechanisms. This biological “chemical plant” operates through tightly coordinated neuroendocrine and metabolic pathways that maintain systemic homeostasis. Throughout scientific history, sustained efforts have been made to unravel the fundamental principles governing these processes; however, their complexity continues to pose challenges long before a comprehensive understanding has been achieved. When any component of this regulatory framework, hormonal, neuronal, or molecular, malfunctions, the effects can propagate across multiple organ systems. Even subtle cellular perturbations may accumulate over time, predisposing individuals to complex pathological states, including psychiatric and metabolic disorders.

Among these interconnected systems, neuroendocrine signaling plays a central role in linking brain function with peripheral metabolism. Dysregulation of the hypothalamic–pituitary–adrenal (HPA) axis has been consistently implicated in the pathophysiology of numerous mental illnesses (1, 2). Contemporary neuropeptidergic models highlight the importance of stress-responsive peptides such as corticotropin-releasing hormone, oxytocin, and vasopressin in modulating emotional regulation, cognitive processing, and social behavior (3). Aberrant activity within these neuroendocrine circuits contributes to the pathogenesis of depression, anxiety, and schizophrenia, partly through their downstream metabolic interactions (4, 5). Neuropeptides, secreted in circadian patterns that are dynamically altered by stress, exert both central and peripheral influences, forming key molecular bridges among mood, cognition, and energy homeostasis.

The high prevalence of comorbidity between psychiatric disorders and metabolic diseases, particularly obesity and type 2 diabetes, underscores a multidirectional disruption of the brain-body axis (6, 7). Meta-analytic evidence indicates that individuals with schizophrenia, bipolar disorder, or major depressive disorder exhibit persistent alterations in glucose regulation, lipid metabolism, and autonomic function, even in the absence of pharmacological confounders (8). Conversely, metabolic dysregulation can amplify neuroinflammatory and oxidative stress pathways, accelerating psychiatric symptom progression and cognitive decline. Despite growing recognition of this bidirectional relationship, clinical management remains suboptimal. Diagnostic biases, social stigma, and the metabolic side effects of psychopharmacological agents continue to obscure accurate assessment and timely intervention.

Recent research extends this concept by linking systemic metabolic imbalance to organ-specific abnormalities in psychiatric populations. Metabolically-dysfunction-associated fatty liver disease (MAFLD), for example, has been identified even in lean individuals with bipolar disorder, indicating underlying metabolic vulnerability irrespective of body mass index. Similarly, autonomic markers such as heart rate deceleration capacity have shown prognostic value in acute ischemic stroke, a condition influenced by both metabolic and neuropsychiatric factors. These observations reinforce the hypothesis that cardiovascular, neuroendocrine, and psychiatric systems form a dynamically regulated continuum rather than discrete entities.

Parallel advances in biomarker research provide new diagnostic perspectives. Altered circulating levels of norepinephrine and melatonin have emerged as reproducible neuroendocrine signatures in schizophrenia, reflecting disruptions in circadian–metabolic coupling. Meanwhile, advances in cardiac imaging, such as two-dimensional speckle tracking echocardiography, have enabled non-invasive assessment of myocardial strain and therapeutic outcomes in patients with combined heart failure and metabolic syndrome, further illustrating the systemic reach of neuroendocrine regulation.

Recent developments in computational modeling and biomarker integration demonstrate the translational potential of multi-parametric diagnostics. Predictive nomograms incorporating blood-derived biomarkers have successfully forecasted short-term recovery outcomes in stroke patients, where neuroendocrine stress reactivity serves as a critical determinant. Collectively, such findings highlight a paradigm shift: psychiatric and metabolic disorders must be conceptualized as interconnected manifestations of a shared neuroendocrine framework rather than as isolated conditions.

Ultimately, elucidating neuroendocrine mechanisms underlying psychiatric–metabolic comorbidity presents an opportunity to redefine both disease classification and therapeutic intervention. Future strategies aimed at modulating neuropeptide signaling, circadian alignment, and stress reactivity hold promise for enhancing mental and metabolic health concurrently. Progress in this field will depend on interdisciplinary collaboration across neurobiology, psychiatry, and endocrinology, coupled with the integration of multi-omic datasets, longitudinal biomarker profiling, and advanced computational modeling to comprehensively characterize the bidirectional dialogue between the brain and body.

## References

[1] Mikulska J Juszczyk G Gawrońska-Grzywacz M Herbet M . HPA axis in the pathomechanism of depression and schizophrenia: new therapeutic strategies based on its participation. Brain Sci. (2021) 11:1298. doi: 10.3390/brainsci11101298, PMID: 34679364 PMC8533829

[2] Pariante CM Lightman SL . The HPA axis in major depression: classical theories and new developments. Trends Neurosci. (2008) 31:464–8. doi: 10.1016/j.tins.2008.06.006, PMID: 18675469

[3] Nemeroff CB Vale WW . The neurobiology of depression: inroads into treatment and new drug discovery. J Clin Psychiatry. (2005) 66:5. doi: 10.1080/138033990513690, PMID: 16124836

[4] Maes M Kubera M Obuchowiczwa E Goehler L Brzeszcz J . Depression’s multiple comorbidities explained by (neuro) inflammatory and oxidative & nitrosative stress pathways. Neuroendocrinol Lett. (2011) 32:7–24., PMID: 21407167

[5] Lopresti AL Maker GL Hood SD Drummond PD . A review of peripheral biomarkers in major depression: the potential of inflammatory and oxidative stress biomarkers. Prog Neuropsychopharmacol Biol Psychiatry. (2014) 48:102–11. doi: 10.1016/j.pnpbp.2013.09.017, PMID: 24104186

[6] Vancampfort D Correll CU Galling B Probst M De Hert M Ward PB . Diabetes mellitus in people with schizophrenia, bipolar disorder and major depressive disorder: a systematic review and large-scale meta-analysis. World Psychiatry. (2016) 15:166–74. doi: 10.1002/wps.20309, PMID: 27265707 PMC4911762

[7] De Hert M Correll CU Bobes J Cetkovich-Bakmas M Cohen DA Asai I . Physical illness in patients with severe mental disorders. I. Prevalence, impact of medications, and disparities in health care. World Psychiatry. (2011) 10:52. doi: 10.1002/j.2051-5545.2011.tb00014.x, PMID: 21379357 PMC3048500

[8] Penninx BW Lange SM . Metabolic syndrome in psychiatric patients: overview, mechanisms, and implications. Dialogues Clin Neurosci. (2018) 20:63–73. doi: 10.31887/DCNS.2018.20.1/bpenninx, PMID: 29946213 PMC6016046

